# The early drift of the Indian plate

**DOI:** 10.1038/s41598-021-90172-z

**Published:** 2021-05-24

**Authors:** Wilfried Jokat, Tabea Altenbernd, Graeme Eagles, Wolfram H. Geissler

**Affiliations:** 1 Alfred Wegener Institute, Helmholtz Centre for Polar and Marine Research , Am Alten Hafen 26, 27568 Bremerhaven, Germany; 2 Geoscience Department, University of Bremen, Klagenfurter Str. 4, 28359 Bremen, Germany

**Keywords:** Solid Earth sciences, Geology, Geophysics, Tectonics

## Abstract

Plate kinematic models propose that India and Sri Lanka (INDSRI) separated from Antarctica by extremely slow seafloor spreading that started in early Cretaceous times, and that a long-distance ridge jump left a continental fragment stranded off the Antarctic margin under the Southern Kerguelen Plateau ^1-3^. Here, we present newly acquired magnetic and deep wide-angle seismic data that require a fundamental re-evaluation of these concepts. The new data clearly define the onset of oceanic crust in the Enderby Basin and off southern Sri Lanka, and date its formation with unprecedented confidence. The revised timing indicates that India and Sri Lanka detached from Antarctica earlier in the east than in the west. Furthermore, no compelling evidence for an extinct spreading axis is found in the Enderby Basin. A refined plate motion model indicates that India and Sri Lanka departed from Antarctica without major rift jumps, but by the action of three spreading ridges with different timings and velocities that must have been accommodated by significant intracontinental deformation.

## Introduction

**Figure 1:**
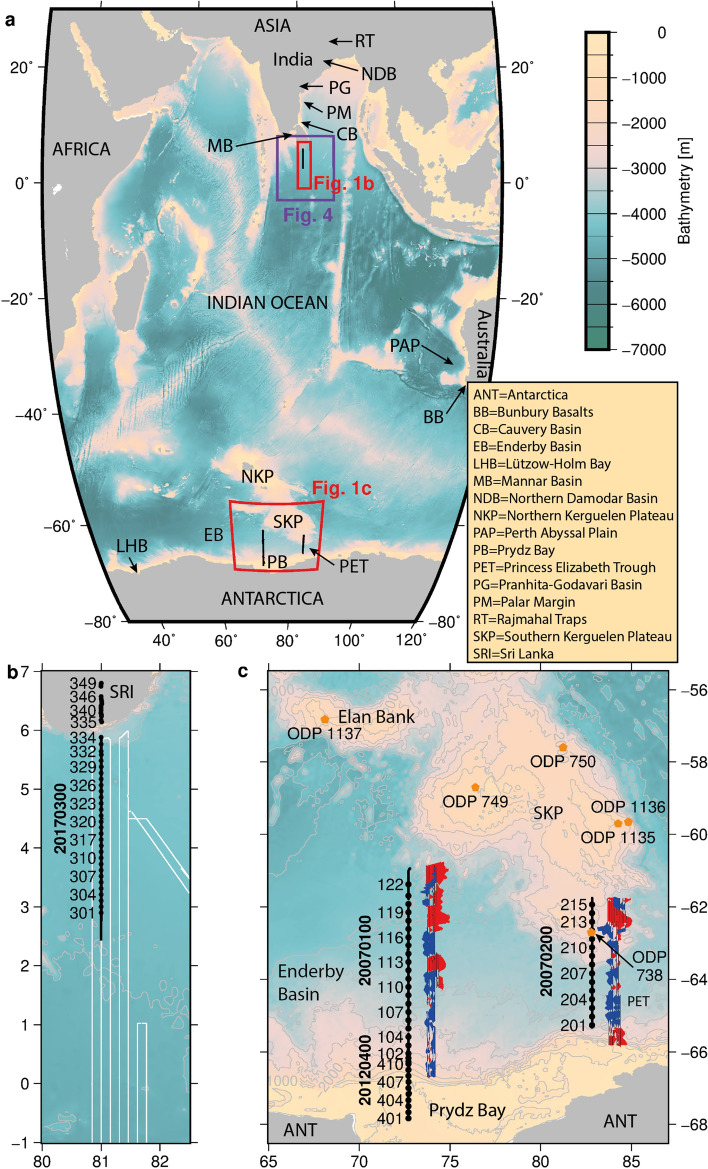
Location of magnetic and seismic profiles in the research areas. Maps: Generic Mapping Tools^36^. (**a**) Overview map showing the location of the research areas in this conjugate margin study. The red and purple boxes indicate the locations of newly-gathered magnetic and seismic data. Black lines mark the locations of newly-acquired seismic refraction data. (**b**) Location of deep seismic line 20170300. Black circles show the positions of the seismic stations on the seafloor and onshore Sri Lanka. White lines show the locations of newly acquired magnetic profiles. (**c**) Locations of the deep seismic profiles in the Enderby Basin and Princess Elizabeth Trough. Black circles represent the locations of OBS and OBH stations. ODP drill sites are marked in orange. Helicopter borne magnetic data (blue and red) were acquired in corridors centred on and parallel to the deep seismic lines. For clarity, they are shown here shifted eastwards.

**Figure 2:**
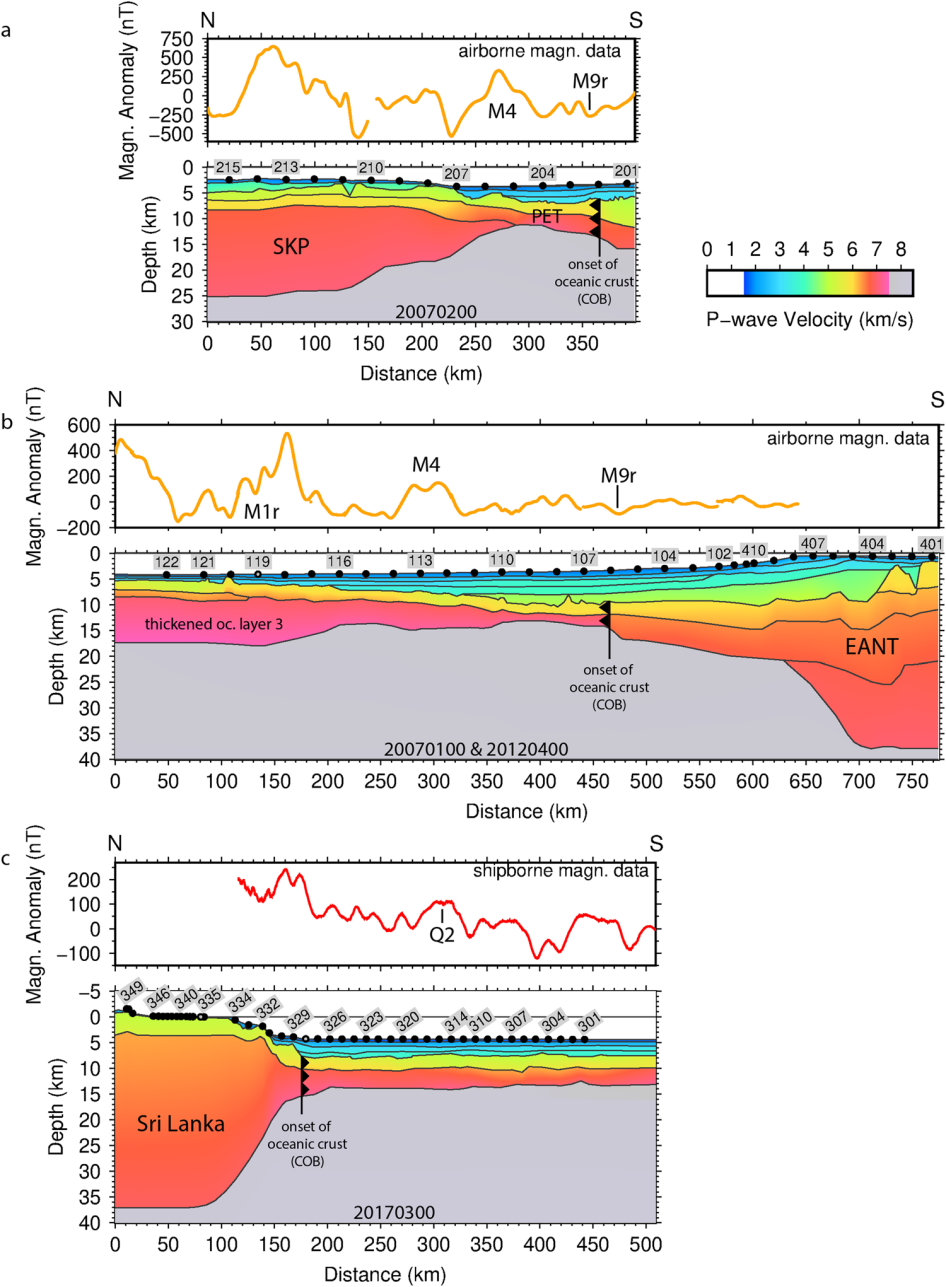
P-wave velocity profiles and magnetic data acquired across the Princess Elizabeth Trough (**a**), off Prydz Bay (**b**), and south of Sri Lanka (**c**). All plots: Generic Mapping Tools^36^. The upper panels display the newly acquired magnetic data along each line, the lower panels the P-wave velocity models. The positions of OBSs, OBHs and land stations are marked by black circles. Lost stations and stations with serious malfunctions are marked by grey circles. Numbers above the stations indicate the OBS/OBH/land station number. Selected magnetic chrons (e.g. M4, M9r) are added to the magnetic data based on identifications from this study. Accordingly, the onset of oceanic crust is marked. Profiles are shown at a common scale. The PET and PB velocity models are aligned at their COBs to highlight the spreading rate differences between them. Details of ray coverage, misfits and uncertainties can be found in the Supplementary Figures S2–S10 and Supplementary Table S11. Further details will be published elsewhere. Abbreviations: COB-continent-ocean boundary (onset of oceanic crust), EANT-East Antarctica, oc-oceanic, PET-Princess Elizabeth Trough, SKP-Southern Kerguelen Plateau.

**Figure 3:**
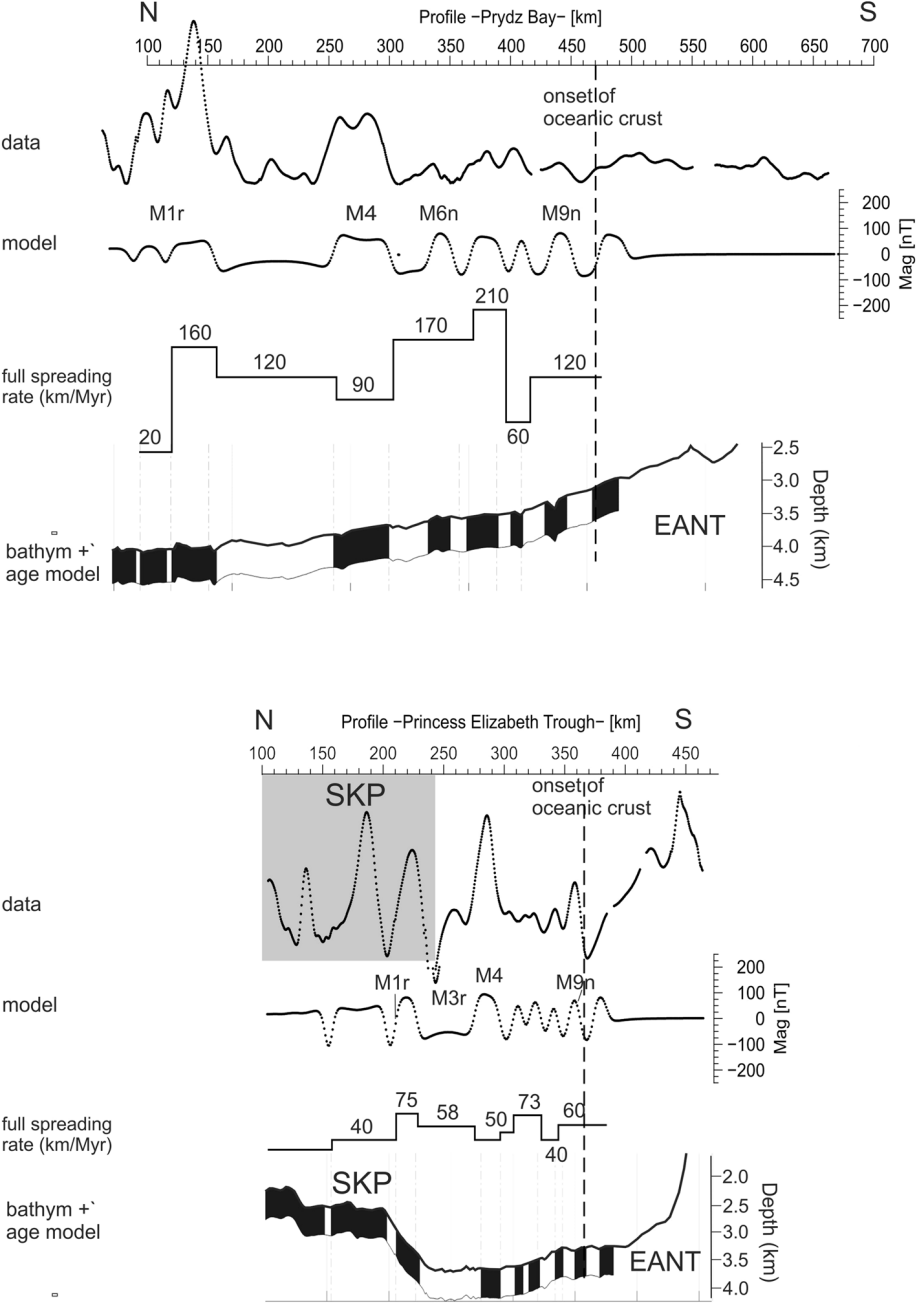
ModMag^32^ models of the helicopter-borne magnetic data off Prydz Bay (upper panel) and in Princess Elizabeth Trough (lower panel). See “Methods” for more details of the modelling. The spreading rates are based on the same timescale models that we use for times and ages given in the text^10,11,14^. Both panels are plotted to the same scale and can be directly compared. The strong magnetic anomalies in the upper panel between kms 100 and 160 are related to shallow basement of the Kerguelen Plateau and have to be interpreted with caution. In the lower panel, almost 240 km of the profile crosses the Southern Kerguelen Plateau (SKP), where shallow basement also raises high-amplitude anomalies. Abbreviations: See Fig. 2.

**Figure 4:**
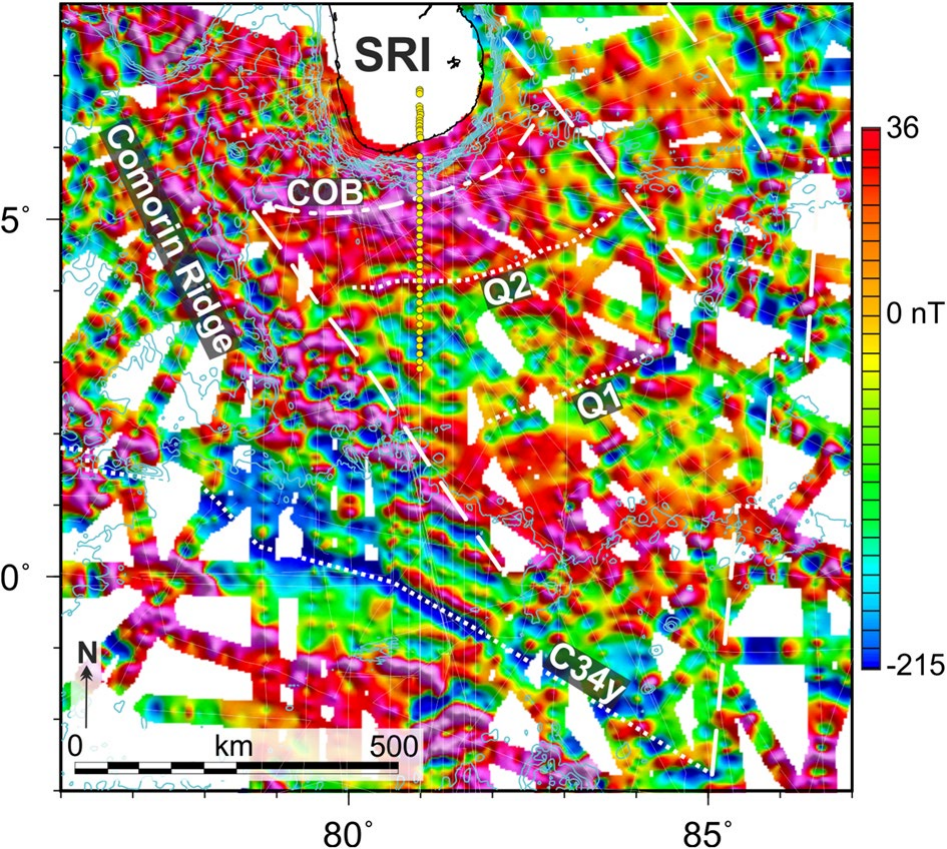
Compilation of our new magnetic data and all available regional shipborne magnetic data south of Sri Lanka (for line coverage see Supplementary Fig. S1). Map: Geosoft Oasis Montaj (https://www.seequent.com/products-solutions/geosoft-oasis-montaj/). Grey lines indicate the locations of newly acquired magnetic data between 5° N and 3° S (Fig. 1b), blue lines are selected bathymetric contours. The line spacing of the new shipborne data is approximately 20 km. Yellow circles show the positions of seismic stations along profile 20170300 (Fig. 2c) both on the seafloor and onshore Sri Lanka. See methods section for details of processing and gridding the magnetic data. Abbreviations: COB-continent-ocean boundary (onset of oceanic crust); Q1, Q2 and C34y—interpreted geomagnetic reversal and fluctuation intensity isochrons, age model according to^10,11,14^.

INDSRI breakup models rely on magnetic seafloor spreading isochron identifications in the Enderby Basin off Antarctica (ANT) and its conjugate region off East India^1–4^. Models using these identifications currently feature two phases of separation. The first phase starts around 136 Ma (chron M11) at a mid-ocean ridge in the Enderby Basin^2,3^. This starting mid-ocean ridge is framed by the continental margins of East Antarctica in the south, and of Elan Bank and southern Kerguelen Plateau (SKP) in the north (Fig. 1). Starting around 121 Ma, the second phase sees this ridge abandoned in favour of a new one further north of Elan Bank and the SKP, which subsequently form the Enderby Basin’s northern continental margin^2,3^. The new mid-ocean ridge, formed by this ridge jump, was located off the eastern continental margin of India. Later, eruptions of magma originating from the Kerguelen mantle plume formed the Rajmahal trap basalts (Fig. 1) around 118–113 Ma on the Indian Plate^5,6^. The principal evidence for the northward ridge jump is the discovery of felsic clasts in a polymict gravel cored during ODP Leg 183, at site 1137 on Elan Bank to the west of the northern Kerguelen Plateau^7^ (Fig. 1, NKP). This gravel is the main reason why Elan Bank is interpreted as a continental sliver that became detached from India by the action of the new mid-ocean ridge along the continent’s eastern margin^2,8^. As well as the Rajmahal eruptions on the Indian plate, ongoing presence of the Kerguelen mantle source led to the eruption of the Kerguelen Plateau (KP) large igneous province at the northern margin of the abandoned Enderby Basin in the Antarctic plate (Fig. 1). According to these two-phase models^1–3^, at least two microcontinents or continental fragments (Elan Bank and SKP) lie embedded in the ocean floor that formed by the divergence of the Indian and Antarctic plates. The Mesozoic seafloor spreading isochrons on which the two-phase model is based are derived from widely spaced and unevenly distributed ship-borne data^2^. Another set of constraints, the locations of the onset of oceanic crust off INDSRI and East Antarctica, are similarly controversial because they are not constrained by any deep seismic data. Previously, dense aeromagnetic data^9^ were collected off western Enderby Land (Fig. 1, Lützow Holm Bay-LHB) to test the two-phase models’ predictions of M-Series isochrons there. These data ruled out the presence of M-series anomalies, and instead showed that the seafloor mainly formed during the Cretaceous Normal Superchron (CNS)^9^. This observation reduced confidence in the two-phase model. In this study, we test further the two-phase model by making use of new magnetic and seismic wide-angle data acquired in the Enderby Basin and south of Sri Lanka (Figs. 1, 2, 3, 4) by one airborne and three ship-based surveys during the last decade. Merging these data with legacy magnetic data allows us to precisely and confidently define the distribution and age of oceanic crust in both regions. These findings do not support a two-phase model.

## Results

### Princess Elizabeth trough transect

Starting at 83°E in the Princess Elizabeth Trough (PET), the deep seismic line 20070200 images the onset of 7 km-thick oceanic crust at km 365 (Fig. 2a). South of this, the landward extent of transitional crust could not be determined because sea ice prevented the deployment of OBSs on the continental shelf. Northwards of its onset, the oceanic crustal thickness gradually thins to 4 km over a distance of 75 km. North of km 290, the crust thickens gradually again to 22 km at the rim of the SKP (Fig. 2a), which we interpret to indicate the delivery of excess melt from the KP mantle plume to the mid-ocean ridge in PET. Dense helicopter magnetic data east and west of this seismic line show clearly-correlatable east-striking spreading anomalies (Fig. 1c). The oldest isochron interpreted from these anomalies is M9r (133.5 Ma^10,11^, Fig. 2a). The onset of thicker crust at km 290 coincides with chron M4 (~ 130 Ma). The full rate of spreading in the PET varies moderately between 40 and 75 km/Myr (Fig. 3, lower panel). Neither data set provides evidence for an extinct spreading centre in PET or continental basement beneath SKP.

### Prydz Bay transect

Approximately 460 km further to the west, off Prydz Bay (PB; Fig. 1), the second P-wave velocity model (profiles 20070100 and 20120400) indicates the onset of oceanic crust approximately at profile km 470 (Fig. 2b). North of this point, the velocity-depth distribution is typical for oceanic crust (see “Methods” section for more details), but the oceanic crust is thin, as little as 3.5 km, or half the global average^12^. Towards the south, the model shows an approximately 230 km-wide zone of transitional crust (km 470 to km 700 ). Continental crust is present south of profile km 700 (Fig. 2b). The crucial result of this experiment is that oceanic crust continues 160 km farther south into PB than previously assumed. The previous assumption, of an onset near km 300, was a leading constraint on the two-phase models (Fig. 2b)^1–3,15,16^, which require the area further south to be floored by transitional or continental crust. To date the seismically-imaged oceanic crust south of km 300 (Fig. 2b), we use dense helicopter magnetic data acquired in a 20 km-wide swath parallel to the seismic profile (Fig. 1c). Modelling indicates that these data portray the presence of a set of M-Series magnetic reversal isochrons (Fig. 3, upper panel). The isochron sequence shows that oceanic crust in the Enderby Basin first formed at chron M9r (133.5 Ma) with a full spreading rate of 120 km/Myr (Fig. 3). North of km 350 (Fig. 2b), the oceanic crust thickens to 12 km near the rim of KP. This thickening is most pronounced in the lower oceanic crust (oceanic layer 3, Fig. 2b), a signature of spreading in the presence of excess melt supply from the Kerguelen plume^13^. The dense magnetic data show that the delivery of excess melt started around chron M4 (130 Ma^11^) (Fig. 3, upper panel), as in PET. Around this time, the full spreading rate dropped from 170–210 km/Myr to 90–120 km/Myr (Fig. 3, upper panel). The set of anomalies in the new magnetic data does not show the symmetry that would support the presence of an extinct spreading axis^2^. Overall, the former mid-ocean ridge in PB accommodated plate divergence at a spreading rate twice as fast as that in PET (Fig. 3). This imbalance must have been accommodated by intra-continental relative movements of 100–155 km magnitude in India and/or Antarctica.

### Sri Lanka transect

The breakup-stages of all two-phase kinematic models of the central Indian Ocean portray Sri Lanka in a location off Lützow Holm Bay (LHB) in East Antarctica (Fig. 1)^1–3,15,16^. With this in mind, new geophysical data were acquired to define the extent of oceanic crust south of Sri Lanka (Fig. 1c) and test some of the assumptions of those models^1–3,15,16^. Critically, these assumptions include previous interpretations of M-series isochrons^3,4^ in sparse magnetic data south of Sri Lanka, which contradict the identification of CNS-age seafloor in much denser data from the conjugate region off LHB^9^. A new wide-angle profile, 20170300, shows the width of transitional crust along the profile south of Sri Lanka is 65 km (Fig. 2c; km 110 to km 175). The onset of oceanic crust at profile km  ~ 175 (Fig. 2c; ~ 90 km off the coast of southern Sri Lanka) is well defined along the profile and by accompanying systematic magnetic data in an approx. 100 km-wide corridor centred on 81° E (Figs. 1, 4). The seismic profile reveals a 5–7 km thick oceanic crust underlying 3–4 km of sediments at 0°/81° E. Here, the magnetic data set reveals a sharp strong negative anomaly that is confidently interpretable as the NW–SE trending isochron C34y (84 Ma), the oldest in a coherent set of late Cretaceous and Cenozoic anomalies that become younger towards the south (Fig. 4). North of this, the magnetic data (Fig. 4) show no correlatable sharp high-amplitude peaks or troughs of the kind that would be expected to mark further reversal isochrons near the equator. Instead, the only correlatable features form a pair of rounded, weak (~ 100 nT), NE-striking lineations, situated ~ 230 km and ~ 480 km south of the coast of Sri Lanka (Fig. 4). Rather than geomagnetic field reversals, these may represent the field-intensity fluctuation isochrons referred to as Q2 and Q1 (108 Ma and 92 Ma)^14^(Fig. 4). Thus, in ruling out the presence of M-series isochrons, the dense magnetic data set off SRI can be understood as a direct conjugate to the one in LHB^9^. With this, the two areas of oceanic crust off SRI and LHB are far younger than predicted by two-phase models of INDSRI-ANT separation.

## Discussion

**Figure 5:**
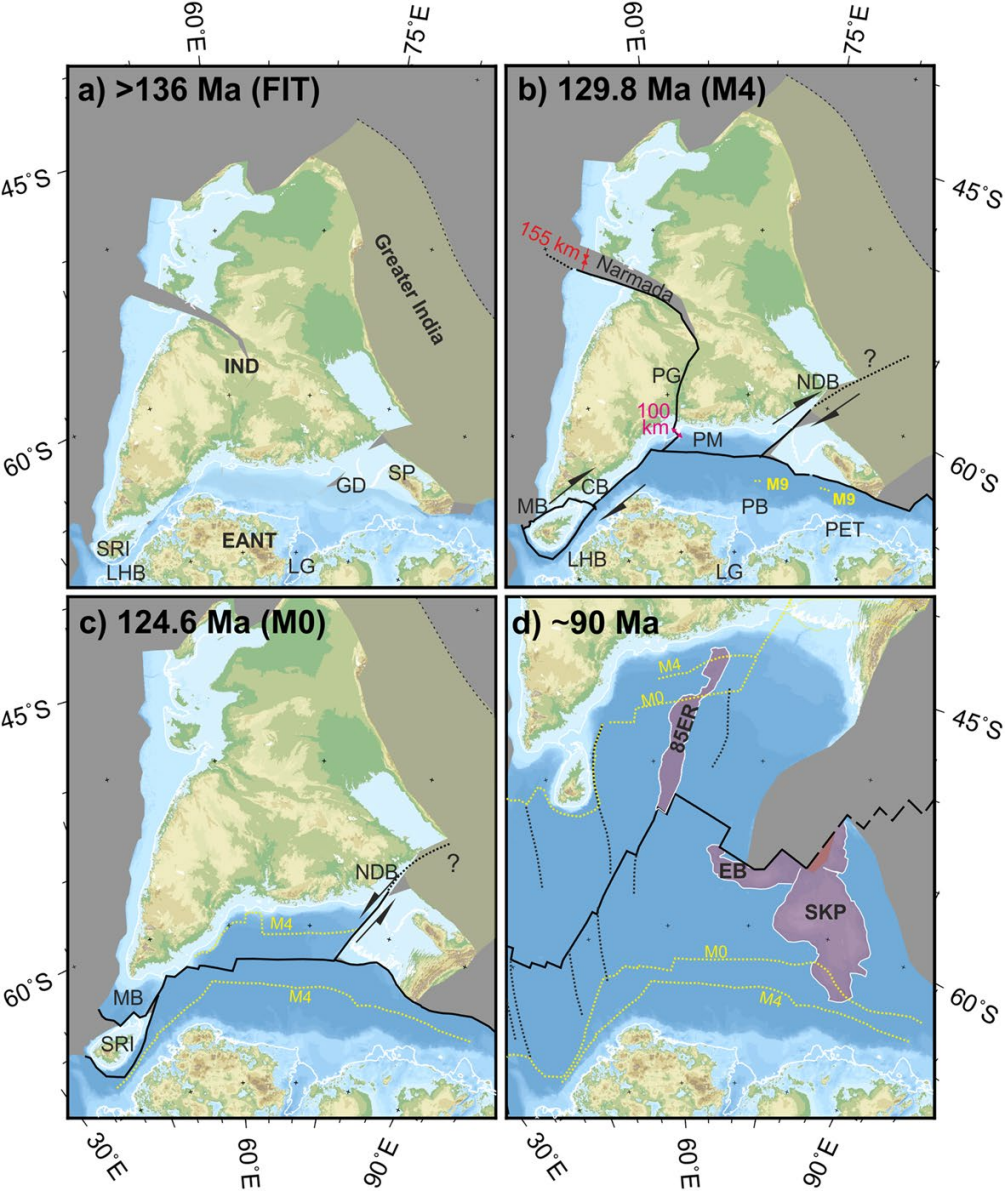
Revised plate kinematic reconstructions. Maps: Generic Mapping Tools^36^. Grey fill: lost area (e.g. Greater India, shortened and/or subducted since Paleogene). Background: present-day topography and bathymetry^33^ (from https://topex.ucsd.edu/cgi-bin/get_data.cgi) and subglacial topography in Antarctica^34^. White outlines: present-day coastlines. Black lines: active plate boundaries. Yellow dotted lines: labelled magnetic isochrons. (**a**) Initial INDSRI-Antarctic fit. Abbreviations: EANT-East Antarctica; GD- Upper Ganges Delta; LG-Lambert Graben; LHB-Lützow Holm Bay; IND-India; SP-Shillong Plateau; SRI-Sri Lanka. (**b**) Chron M4 (~ 130 Ma) reconstruction. Differing rates of early (post-M9/ ~ 133.5 Ma) seafloor spreading in Prydz Bay (PB) and Princess Elizabeth Trough (PET) and slow oblique continental extension in LHB are accommodated by deformation within the Pranhita–Godavari (PG), northern Damodar (NDB), and eastern offshore Palar Margin, Cauvery, and Mannar basins (PM, CB, MB). Red text: maximum estimates of this deformation. (**c**) Reconstruction at chron M0 (~ 125 Ma). New seafloor may have formed within MB. Intracontinental motion within India had ceased except along the NDB, where the sense of strike-slip had reversed. (**d**) Reconstruction at 90 Ma. The Shillong Plateau (labelled SP in **a**) reached its present-day location with respect to India by NDB motion that ended around this time. A major regional plate reorganization saw directions of seafloor spreading change, producing prominent bends in the orientations of fracture zones (thin dotted black lines). Excess volcanism in the M0 to 90 Ma period led to the formation of large igneous provinces at Elan Bank (EB) and the Southern Kerguelen Plateau (SKP), and along the 85° E Ridge (85ER).

The new seismic wide-angle data along all three profiles confidently locate the onsets of oceanic crust off the rifted margins. The profiles in the Enderby Basin image as much as 160 km more oceanic crust than assumed for, and implied by, two-phase models of regional plate motions. Moreover, our dense helicopter magnetic data in the basin allow strongly different and more confident identifications of magnetic reversal isochrons than used in those models^2,15^. These new findings make it necessary to revise kinematic models for INDSRI breakup. Figure 5 shows reconstruction maps illustrating the revised model.

Initially, around 133 Ma (chron M9n), thin (4–7 km) oceanic crust formed at slow (40–60 km/Myr) rates in PET (Fig. 3, lower panel). Spreading at similar rates (70 km/Myr) also started in this period off NE India (Greater India), where it led to opening of the Perth Abyssal Plain (Fig. 1a; PAP) that continued until 101–103 Ma (during the CNS)^16^. Breakup at the PAP was accompanied by eruption of the Bunbury Basalts in SW Australia (Fig. 1a, BB) in three phases (at 134, 133, and 130 Ma)^17^. Compared with the PAP, spreading in PB was much faster around 130 Ma, at 90–210 km/Myr and at highly variable rates (Fig. 3). In LHB, at the same time, plate divergence was accommodated by very slow oblique crustal extension. No oceanic crust formed (Fig. 5b). For our new model, the contrasting rates of continental extension and seafloor spreading in PET, PB and LHB require relative motions of four sub-plates in the interior of INDSRI. This is consistent with fragmentary and sometimes contradictory evidence for Mesozoic tectonic activity onshore in the Pranhita-Godavari^18^ and northern Damodar basins^19^, and offshore tectonic activity at the eastern Palar Margin, and in the Cauvery and Mannar basins^20–22^ (Figs. 1, 5). Our reconstructions provide maximum estimates of these sub-plate motions. The estimates would be reduced by the addition of further sites of intracontinental deformation, for example along the Lambert Graben in Antarctica (Fig. 5b).

Sri Lanka was the final continental part of the Indian plate to separate from Antarctica (Fig. 5c). The identification of Q2 approximately 760 km NW of chron C34y (Fig. 4) implies late-CNS half spreading rates of around 30 km/Myr that are consistent with the relatively smooth oceanic basement surface. At this rate, the 150 km-wide swath of oldest oceanic crust that lies between the continent-ocean boundary south of Sri Lanka and anomaly Q2 (Fig. 4) would date to ~ 112 Ma.

The unusually thin earliest oceanic crust in PET and PB (Fig. 2) indicates a magma-poor environment during initial formation of the Enderby Basin. This is consistent with the absence of evidence for widespread breakup-related magmatism further west in LHB and off INDSRI.

In the next phase of INDSRI’s northward drift, the Kerguelen Plume started to interact with the spreading centres off Enderby Land. Around 130 Ma (chron M4), both the PET and PB seismic transects (Fig. 2) reveal the onset of stepwise northward thickening of the oceanic crust over a 3 Myr period (until 127 Ma/M1r). We relate this observation to the delivery of excess melt to the active mid-ocean ridges with the arrival of the KP mantle plume. While oceanic layer 3 thickened stepwise by a factor of 5–6 at 130–127 Ma (Fig. 2), the first documented eruption of KP plume magma, at the SKP, dates from around 120–110 Ma^23^. During this period, our model suggests that plume material migrated under the Indian plate, giving rise to melt production responsible for the eruption of the Rajmahal Traps (113–118 Ma) and seaward dipping basalt flows below the Bangladesh plains^4,24^. Our data thus support previous suggestions^23^ that the Kerguelen plume did not trigger INDSRI breakup.

For times younger than chron M4, our kinematic model differs strongly from most previous scenarios in not featuring a second phase of spreading that starts with a northward ridge jump. This is firstly because our deep seismic data do not support the presence of a sliver of detached continental crust where they cross onto the SKP (Fig. 2a). Secondly, it is because our dense helicopter magnetic data do not show the symmetrical anomalies that would be expected to mark the flanks of an extinct spreading centre^2^. We note that previous suggestions of continental velocities under Elan Bank were based on wide-angle data from a profile oriented oblique to the proposed continent-ocean boundary, with receivers that were too-widely spaced to determine a reliable seismic velocity profile across the Elan Bank margins^8^. The geochemically-estimated 5% contamination of SKP lavas by continental material^25^ may be more simply explained by incorporation of Gondwana lithospheric material into the plume source from the Antarctic margin just 70 km further south^26^ than by intrusion of plume melt into a continental SKP.

Extrapolation of the late-CNS full spreading rates of ~ 60 km/Myr south of Sri Lanka suggests that the INDSRI-Antarctic mid-ocean ridge system had only propagated to the LHB sector at the western end of the ANT-INDSRI plate boundary by ~ 112 Ma (Fig. 5). Similar CNS rates were extrapolated within the LHB sector using aeromagnetic data^9^. These rates are, in turn, comparable to the rate of northward propagation in Kerguelen LIP eruption ages (40–50 km/Myr)^23^ that record the Indian plate’s northwards progress over the underlying mantle. The arrival of the KP mantle plume thus seems to have ushered in a phase of continuous and consistently moderately-fast northward motion of a single Indian plate.

In summary, our new data provide new constraints on the timing and geometry of early INDSRI-Antarctica plate divergence. The contrasting spreading rates at mid-ocean ridges in the PB and PET sectors imply intracontinental deformation of India and/or Antarctica that in turn makes it possible to reunite the revised continent-ocean boundary locations much more tightly than in the previous two-phase models. This intracontinental relative motion, on the order of 100–155 km, now remains to be proved independently (Fig. 5b). The thin earliest and thicker younger oceanic crust indicates how a magma-poor environment during the initial drift phase was transformed by the delivery of melt from Kerguelen plume mantle around 130 Ma. Based on this, the arrival of the Kerguelen plume did not trigger plate separation. Prior to M4, in a period during which the plume can be expected to have been ascending through the upper mantle in the region, INDSRI motion in both PET and PB shows short-lived accelerations over chrons M7-M6 (Fig. 3). Finally, Sri Lanka, at the southern continental tip of INDSRI, separated from Antarctica by the latest at around 112 Ma. INDSRI’s subsequent more or less continuous northward drift did not feature major rift jumps, and so left no regional extinct spreading axes in the Enderby Basin.

## Methods

The magnetic anomaly grid in Fig. 4 was calculated from along-track data recorded by ships in the region around Sri Lanka and eastern and southern India (supplementary Fig. S1). Of these, the towed magnetometer used for the INGON cruise in 2017 returned 10,780 line-kilometres of new data^27^. These were processed together with the remaining 130,330 km of legacy data downloaded from the NCEI (formerly NGDC) trackline database (https://maps.ngdc.noaa.gov/viewers/geophysics/). All processing was completed using tools in Seequent’s *Geosoft Oasis Montaj* software (URL: https://www.seequent.com/products-solutions/geosoft-oasis-montaj/).

The magnetic field data were generated over a period of six decades by multiple institutions and working groups using a wide range of equipment and acquisition parameters and procedures, and have experienced differing processing histories. It would be prohibitively difficult or impossible to reconstruct these details with the intention of accounting for them by reprocessing. It can be assumed that the data have not undergone meaningful diurnal correction because of the long distances to land, where any base station magnetometer or observatories could be deployed to generate the necessary data. The effects of all this are evident at 1528 cross-point, at which errors reach values as large as 733 nT with a mean value of 185 nT and standard deviation (﻿σ) of 143 nT. From this it is evident that the profiles must be brought to a common level before gridding for visual interpretation.

Because of the lack of any obvious population of tracks with mutually-consistent long-wavelength components to anchor the levelling process, we levelled the data to MF7. MF7 is a long-wavelength (> 300 km) representation of the lithospheric magnetic anomaly field, based on data from the 2007–2010 CHAMP satellite mission^28^ (http://www.geomag.org/models/MF7.html). For each track segment in the ship-based data set, we calculated the along-track differences to MF7. We filtered these differences at lengths less than or equal to the resolution of MF7 using a simple Gaussian filter, and then subtracted the filtered along-track difference from the measured along-track field variation. The effect of this is to replace long wavelengths in the measured data with those of the MF7 field. The choice of Gaussian filter is made on the basis of a trade-off between the self-consistency and waning power of MF7 at wavelengths decreasing towards its cutoff at 300 km, and the retained power but relative inconsistency of ship-track data at wavelengths increasing towards the cutoff. Guided by cross-over error analyses of the adjusted data (N: 1491, Max: 309 nT, Mean: 34 nT, σ: 37 nT) and visual assessment of grids calculated from them, we chose a Gaussian filter of 180 km length. In practice, this means that the magnetic anomaly grid in Fig. 4 retains no information in the 180–300 km wavelength band, but retains all wavelengths in the sub-180 km band in the form they appeared in the ship data. These characteristics mean the grid is suitable for interpreting the edges of magnetic reversal anomalies in oceanic crust, which at the low latitudes of the study area appear as linear narrow high-amplitude anomaly peaks and troughs.

For the modelling of the seafloor spreading anomalies/velocities, we used a modified version of the *ModMag* software^32^. A 0.5 km-thick magnetized layer was assumed with its top surface at the present seafloor. Its magnetization was fixed to 5 A/m. The mean latitude of the two profiles off East Antarctica during the formation of the initial ocean crust was assumed at 70° S. The inclination of the Early Cretaceous magnetic field was set to − 67° in PB and − 72.6° in PET, and its declination to − 69.6° in PB and − 73° in PET. We assumed symmetrical spreading at all times for calculating the full spreading rates given in the text.

### Seismic wide-angle data acquisition and modelling

The three seismic P-wave velocity models presented in this paper are based on data acquired during three different scientific cruises (Supplementary Figs. S2–S10; Supplementary Table S11). Profiles 20070100 and 20070200 along the East Antarctic margin were acquired during a joint expedition in 2007 with RV Akademik Alexandr Karpinskiy and RV Polarstern. Profile 20120400 is a prolongation of profile 20070100 and was acquired with RV Akademik Alexandr Karpinskiy in 2012. Data for 20170300 south of Sri Lanka were collected with RV Sonne in 2017. Coincident multichannel seismic (MCS) and wide-angle seismic data were acquired along profiles 20070200 (PET), 20070100 and 20120400 (PB).

Deep crustal seismic refraction data were collected with OBS (ocean bottom seismometers), and partially also with OBHs (ocean bottom hydrophones) or land stations on Sri Lanka (Supplementary Table S11). All OBSs were equipped with a 3-component seismometer or a geophone, the OBHs with a hydrophone, and all land stations with geophones. More details can be found in the cruise reports^27,29^.

The raw data were archived after recovery of the land- and sea-based seismic recording stations. Time corrections for the drift of internal clock of the recorder were applied where necessary. Raw data from all stations were converted to segy format. The seafloor positions of all marine stations (OBS/OBH) were relocalized using direct wave arrivals. The source-receiver ranges were calculated and written into the segy header.

Refracted and reflected P-wave arrivals were picked with the software *zp* (Barry Zelt, http:// www.soest.hawaii.edu/users/bzelt/zp/zp.html). A bandpass filter of 4–15 Hz and AGCs of 0.7–1 s was applied for picking the first arrivals of different phases. The picked refracted and reflected phases were identified based on their amplitudes, curvatures, and velocities.

For all three models presented here, P-wave velocity-depth modelling was carried out with the forward modelling software *rayinvr*^30^ and the graphical interface *PRay*^31^. By forward modelling, the travel times were fitted with a top to bottom approach. MCS data were used to constrain sedimentary layering and the top basement surface for the P-wave velocity starting model. For the profile south of Sri Lanka, along which no coincident MCS data were gathered, the basement topography was extracted from the OBS data alone. Seismic velocities in the sedimentary and crustal layers were calculated from relevant phases in the seismic refraction data. Layer boundaries were based on picked reflections within the seismic refraction and MCS data. All this information was incorporated into the seismic velocity-depth models. One example of a seismic record section with picks and computed travel times for each profile is shown in Supplementary Figures S2–S10. The position of the onset of oceanic crust was determined by comparing our velocity depth distribution at evenly-spaced locations to global compilations for oceanic^12^ and continental crust^35^. This is the usual approach to define crustal-type boundaries in seismic refraction models.

### Uncertainties and errors

Supplementary Table S11 summarizes the number of picks used for modelling, the average RMS travel time misfits and the χ^2^ values for the three P-wave velocity models. The assigned pick uncertainties for the different phases increase with depth (Supplementary Table S11). Based on these uncertainties, the χ^2^ value for the models ranges between 0.8 and 0.9, which is close to the ideal value of 1. The ray coverage and the travel time picks and computed travel times are shown in Supplementary Figures S2–S10. In the P-wave velocity model of profiles 20070100 and 20120400, the data quality south of profile km 500 is partly poor, which results in a sparse ray coverage for the Antarctic continental margin (Supplementary Fig. S9) and poor resolution in this part of the model.

## Supplementary Information

Supplementary Information.

## Data Availability

The wide-angle data analysed during this study are not fully publicly available, before they are not published, but are available from the corresponding author on reasonable request. The marine magnetic data, and its references to the raw data sets are available through the PANGAEA archive (https://www.pangaea.de/).
