# Supplementary material for: The early drift of the Indian plate
Source: Sci Rep. 2021 May 24;11:10796. doi: 10.1038/s41598-021-90172-z (PMC8144565; doi:10.1038/s41598-021-90172-z)
Supplement: Supplementary file 1 — Supplementary Information. [file 41598_2021_90172_MOESM1_ESM.pdf]

Supplementary Information

The early drift of the Indian plate

Wilfried Jokat, Tabea Altenbernd, Graeme Eagles, Wolfram H. Geissler

Alfred Wegener Institute, Helmholtz Centre for Polar and Marine Research, Am Alten Hafen 26, 27568 Bremerhaven, Germany

(1) Map showing the ship borne magnetic data used in the study
(2) Modelling results and data example for line 20170300; Sri Lanka
(3) Data example and modelling results for seismic profile 20070200,
Princess Elisabeth Trough, East Antarctica
(4) Data example and modelling results for seismic profile 20070100 and 20120400,
Prydz Bay, East Antarctica
(5) Summary Table on deep seismic data acquisition and modelling

(1) Map showing the ship borne magnetic data used in the study

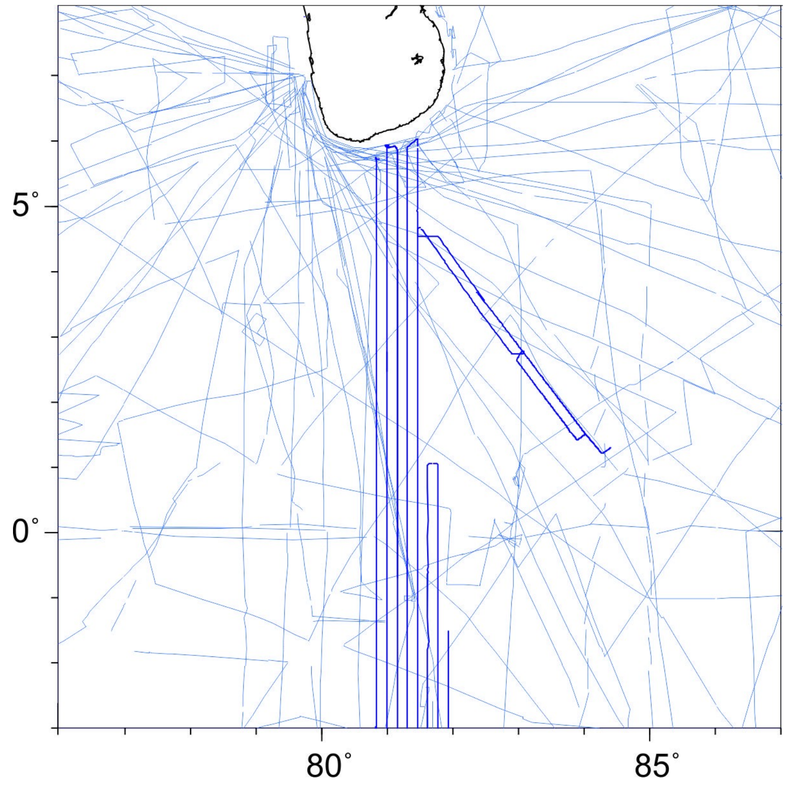

Supplementary Figure S1: Data used for the magnetic total field grid in Figure 2a. Dark blue lines show new magnetic data collected by RV Sonne in 2017. Light blue lines show all other data used, downloaded from the NCEI database (https://maps.ngdc.noaa.gov/viewers/geophysics/) in 2018.

(2) Modelling results and data example for line 20170300, Sri Lanka

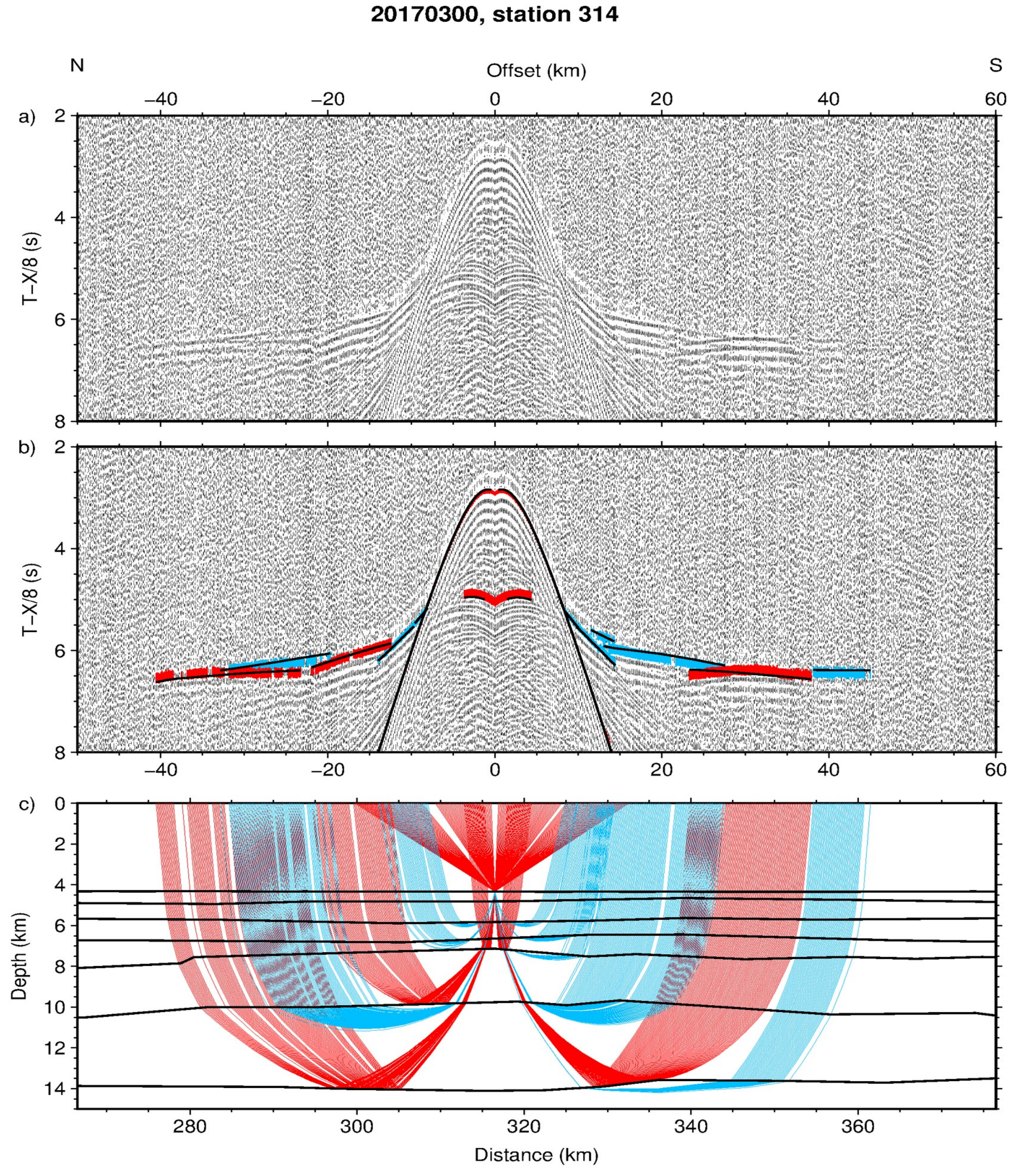

Supplementary Figure S2: Data example for line 20170300.

(a) filtered OBS data

(b) applied travel time picks

(c) raytracing results for this station. Blue: refracted rays, red: reflected rays, black lines: boundaries between velocity layers

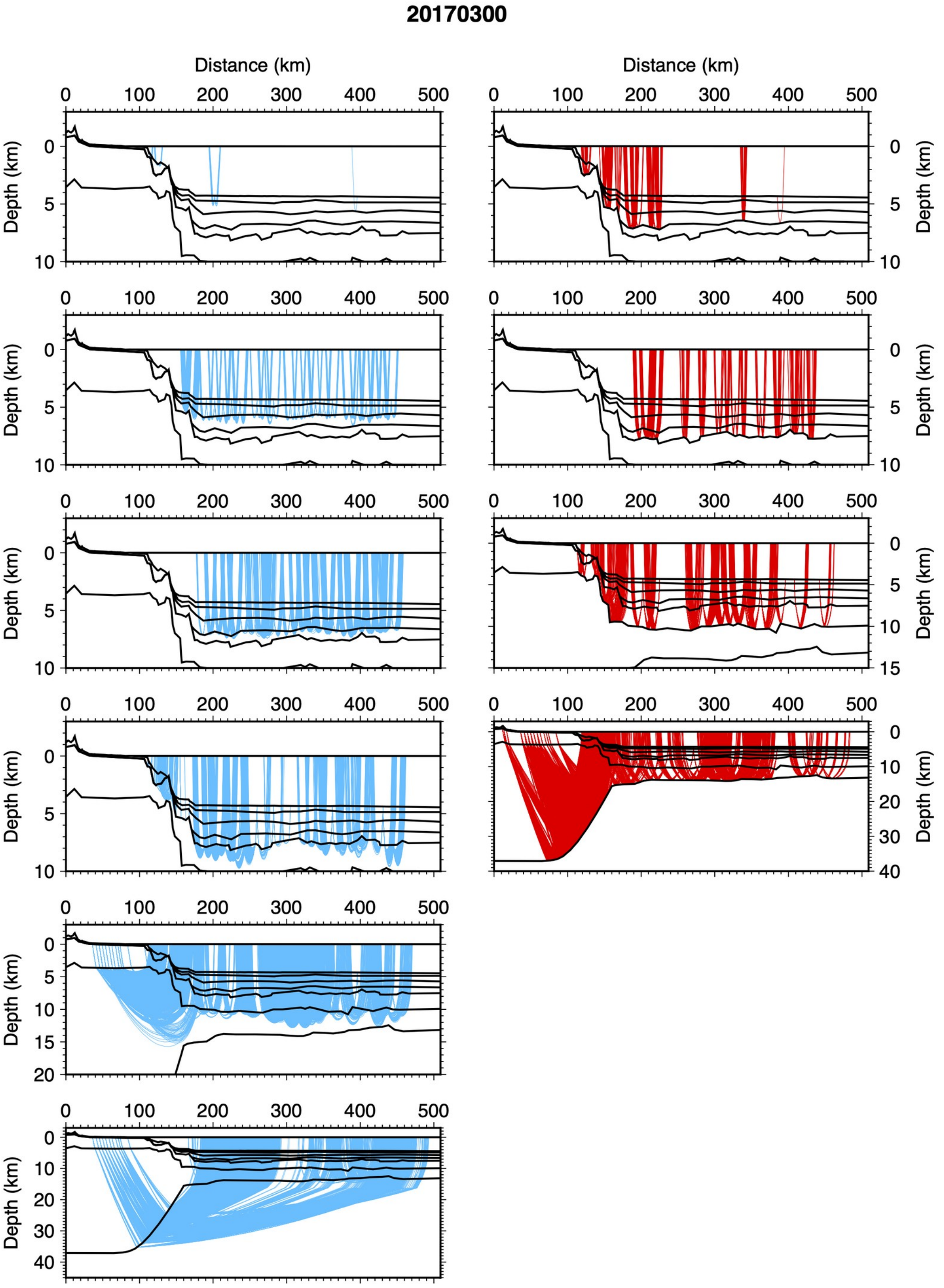

Supplementary Figure S3: Ray coverage of profile 20170300. Blue: refracted arrivals, red: reflected arrivals.

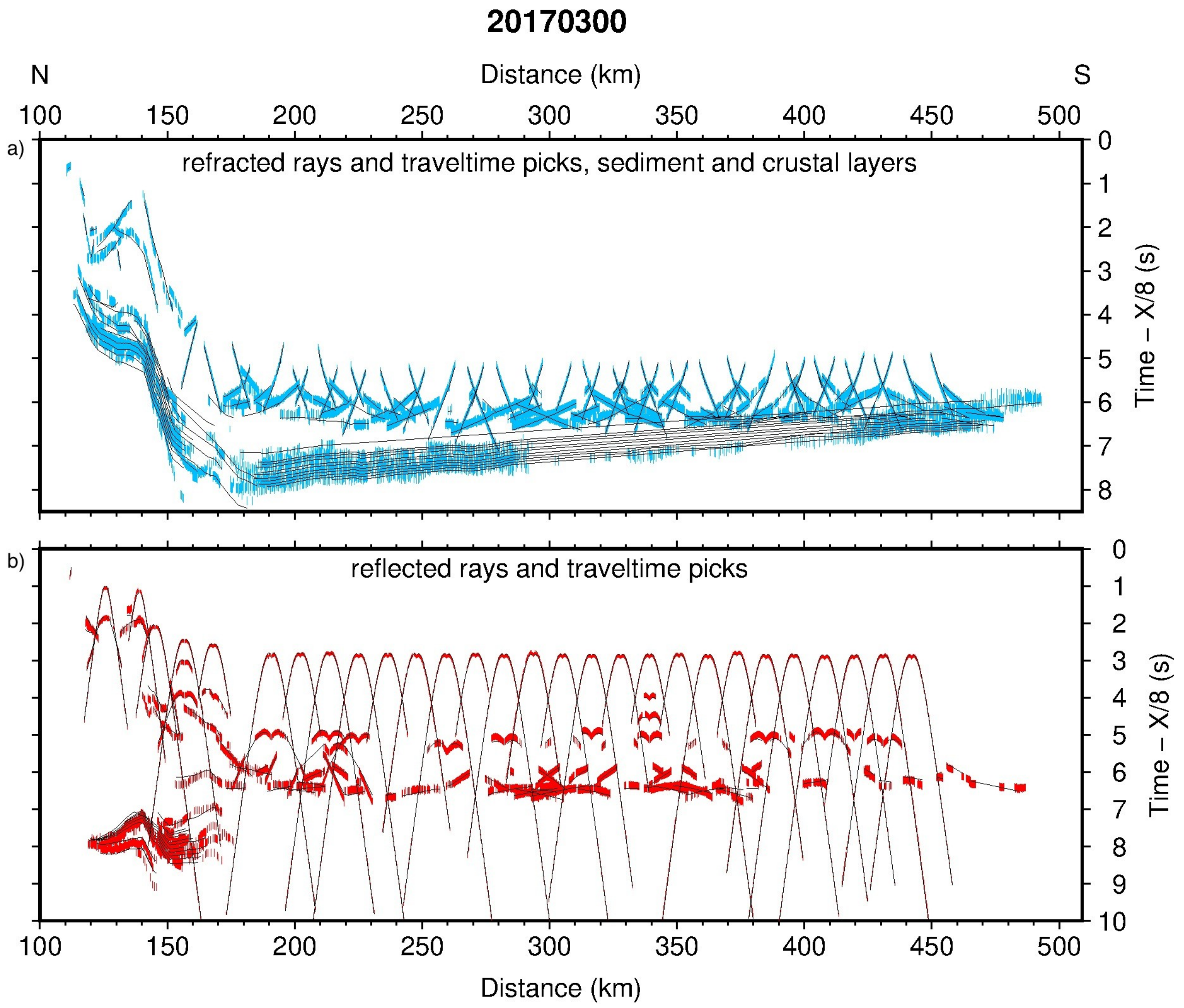

Supplementary Figure S4: Quality of modelled picks for line 20170300

(a) the blue bars show the picks of the refracted arrivals with their error bars

(b) the picks of the reflected arrivals with their error bars are shown in red

For both, the reflected and refracted picks, their length represents the assigned travel time error. Black lines represent modelled travel times.

(3) Data example and modelling results for seismic profile 20070200, Princess Elisabeth Trough, East Antarctica

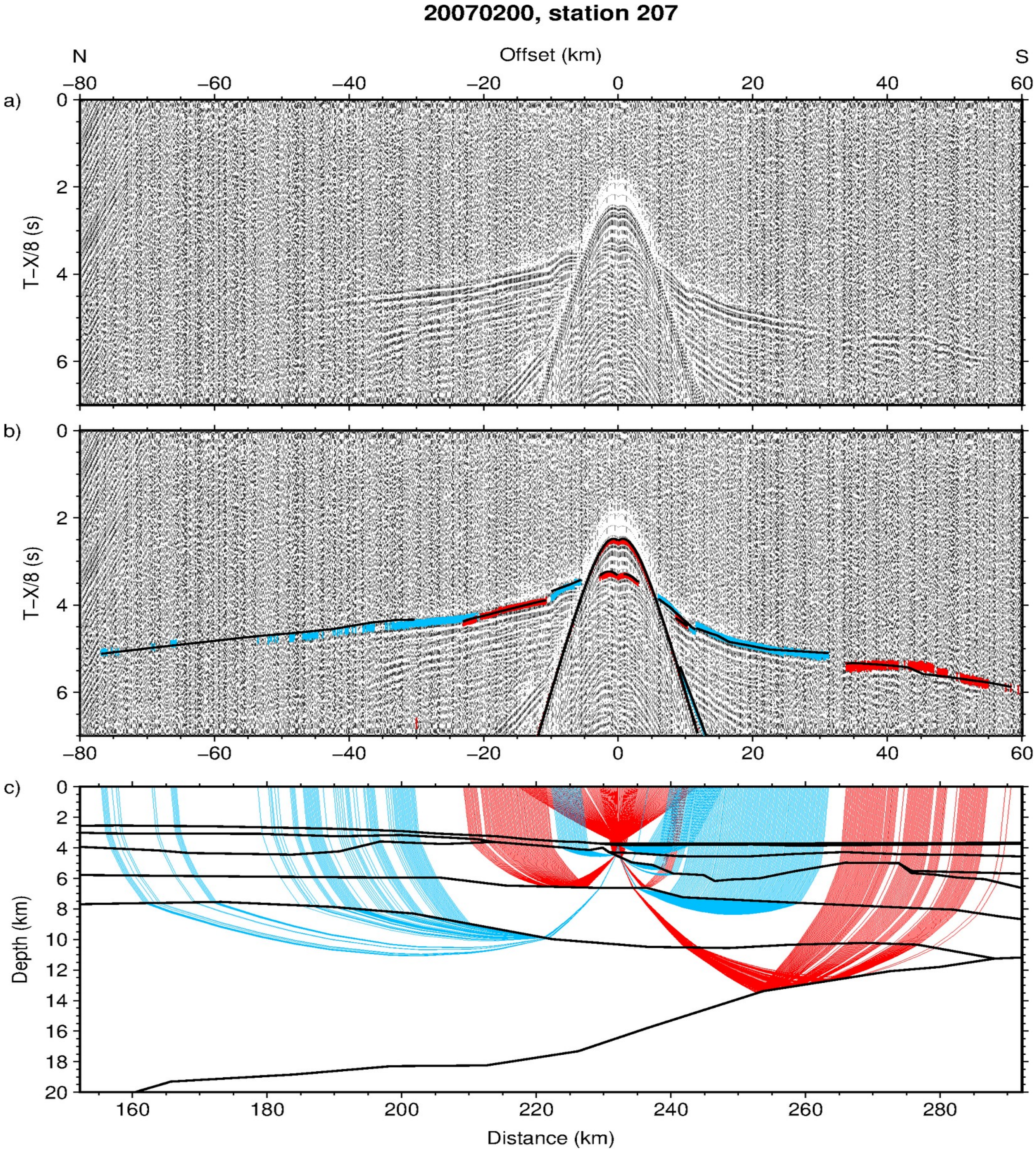

Supplementary Figure S5: Data example for seismic profile 20070200

a) Seismic record section for OBS 204

b) Seismic record section with travel time picks. Blue: refracted arrivals, red: reflected arrivals, black lines: calculated travel times

c) Ray tracing results for this station. Blue: refracted rays, red: reflected rays, black lines: boundaries between velocity layers

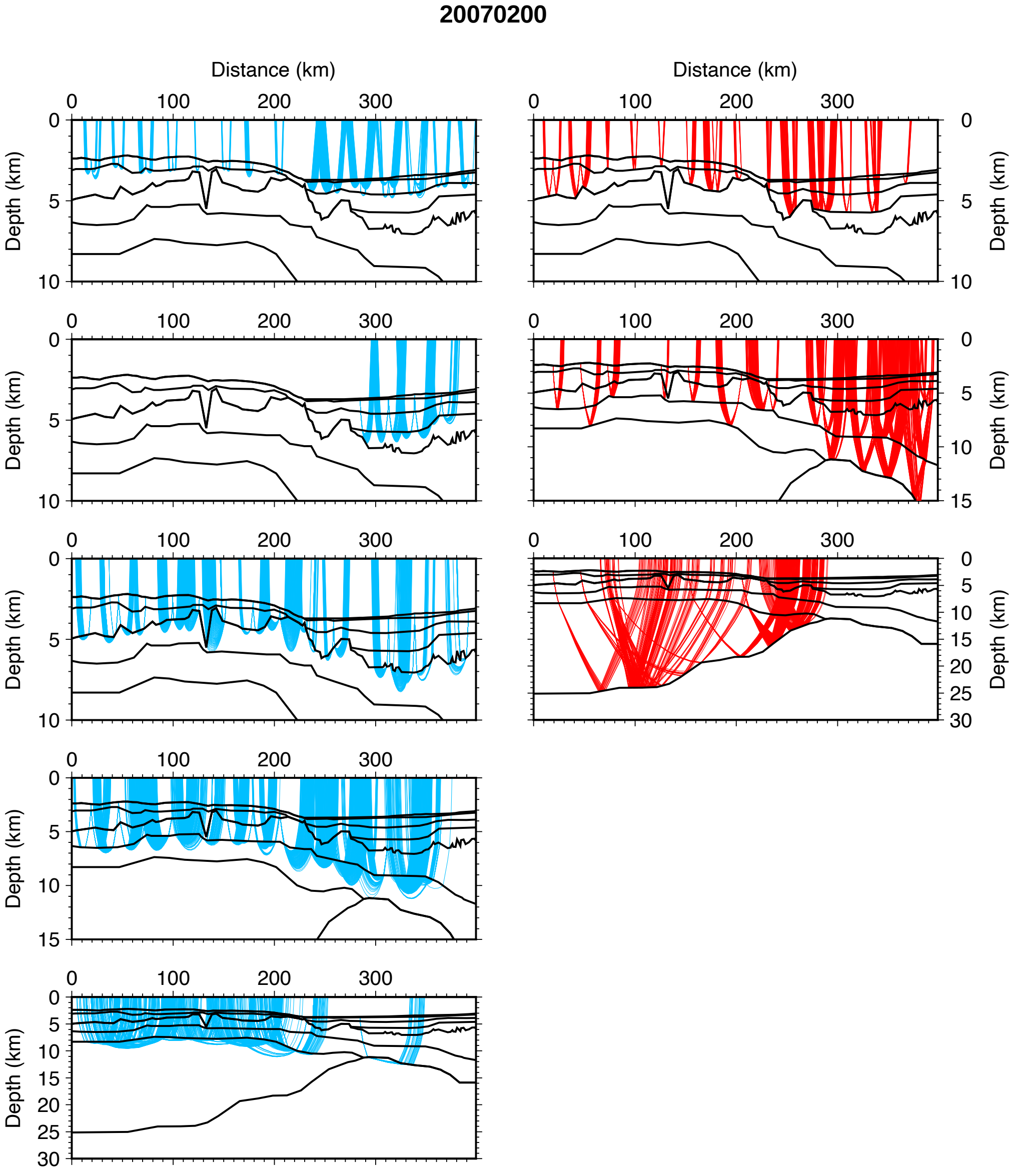

Supplementary Figure S6: Ray coverage of profile 20070200. Blue: refracted arrivals, red: reflected arrivals

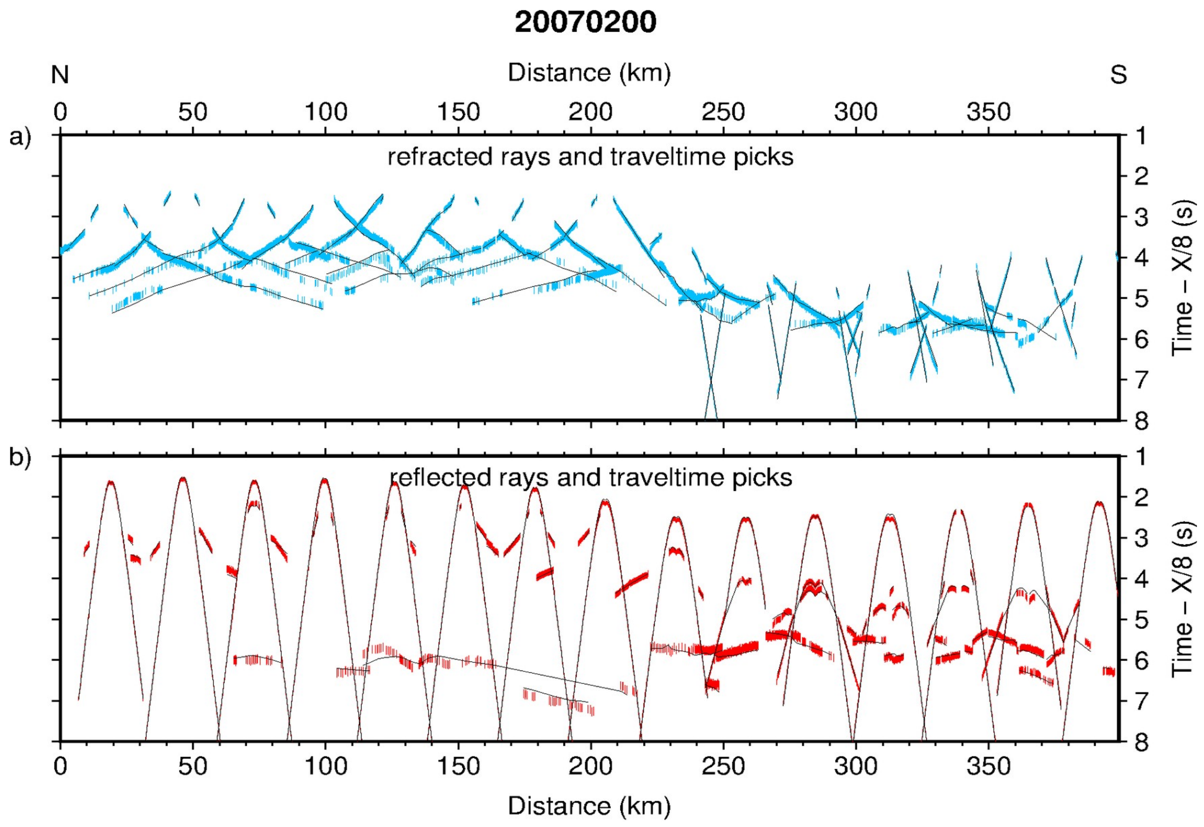

Supplementary Figure S7: Quality of modelled picks for line 20070200

(a) the blue bars show the picks of the refracted arrivals with their error bars

(b) the picks of the reflected arrivals with their error bars are shown in red

For both, the reflected and refracted picks, their length represents the assigned travel time error.

Black lines represent modelled travel times.

(4) Data example and modelling results for seismic profile 20070100 and 20120400 Prydz Bay, East Antarctica

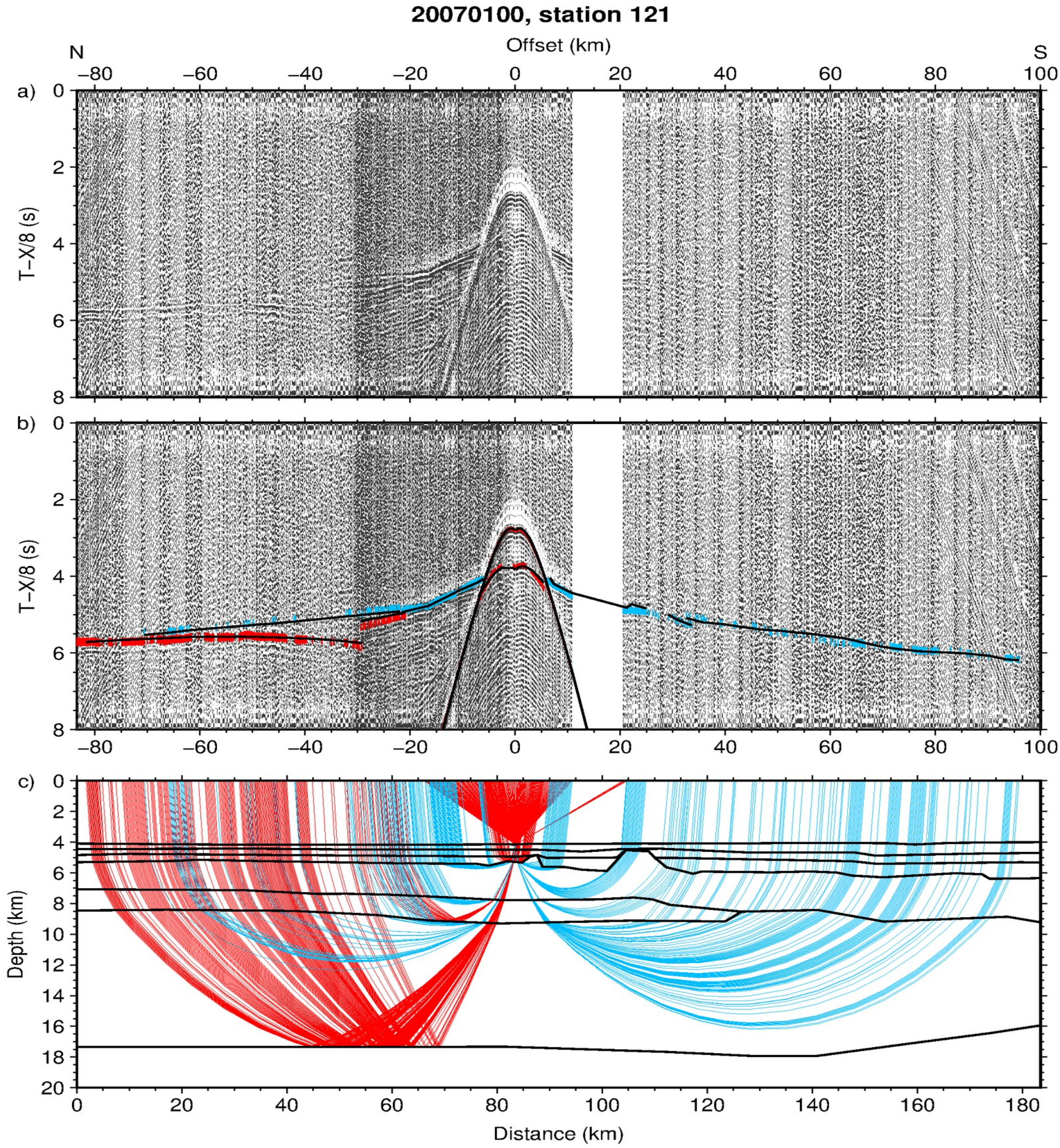

Supplementary Figure S8: Data example for seismic profile 20070100

a) Seismic record section for OBS 121

b) Seismic record section with travel time picks. Blue: refracted arrivals, red: reflected arrivals, black lines: calculated travel times

c) Ray tracing results for this station. Blue: refracted rays, red: reflected rays, black lines: boundaries between velocity layers

20070100 & 20120400

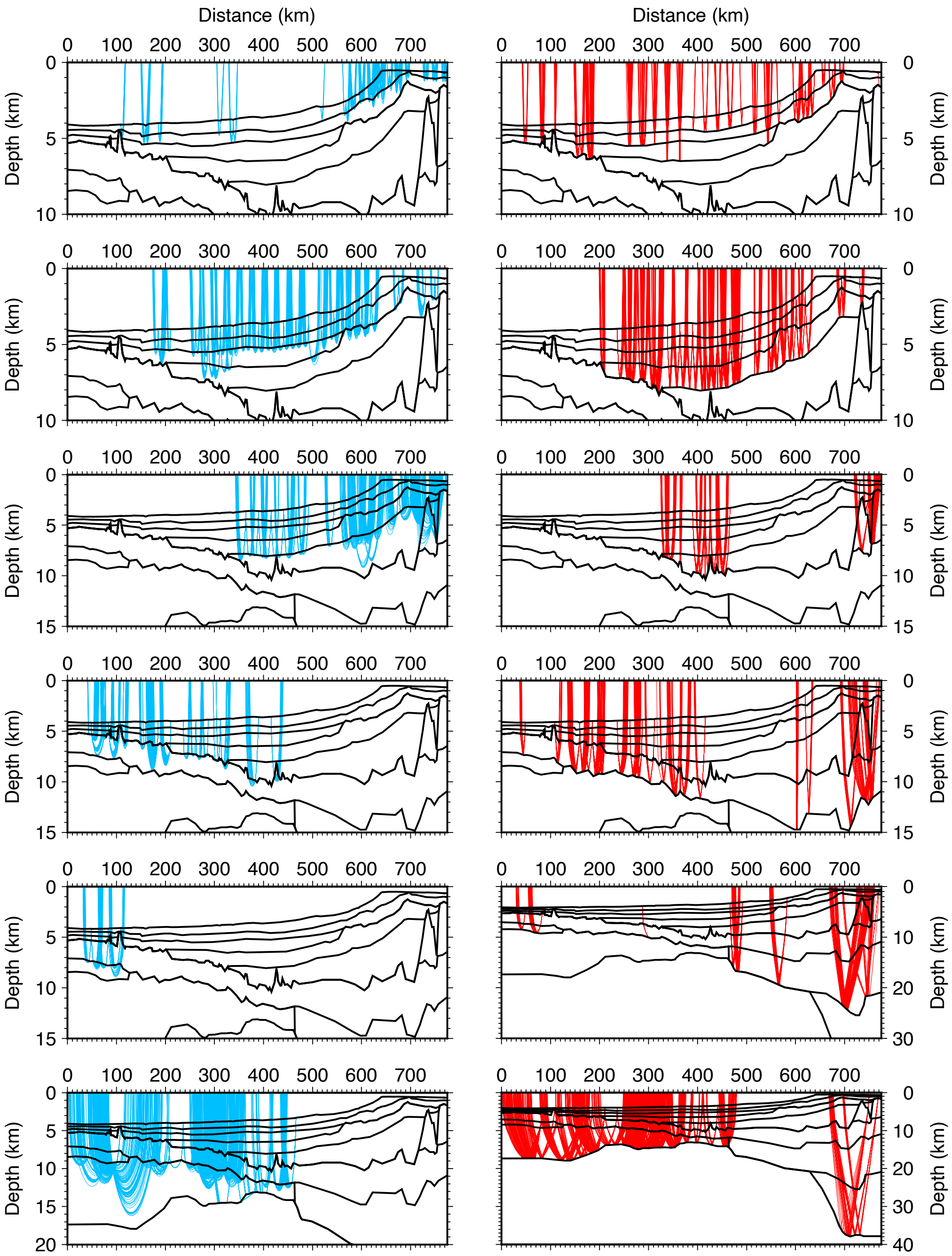

Supplementary Figure S9: Ray coverage of the merged profiles 20070100 and 20120400.

Blue: refracted arrivals, red: reflected arrivals

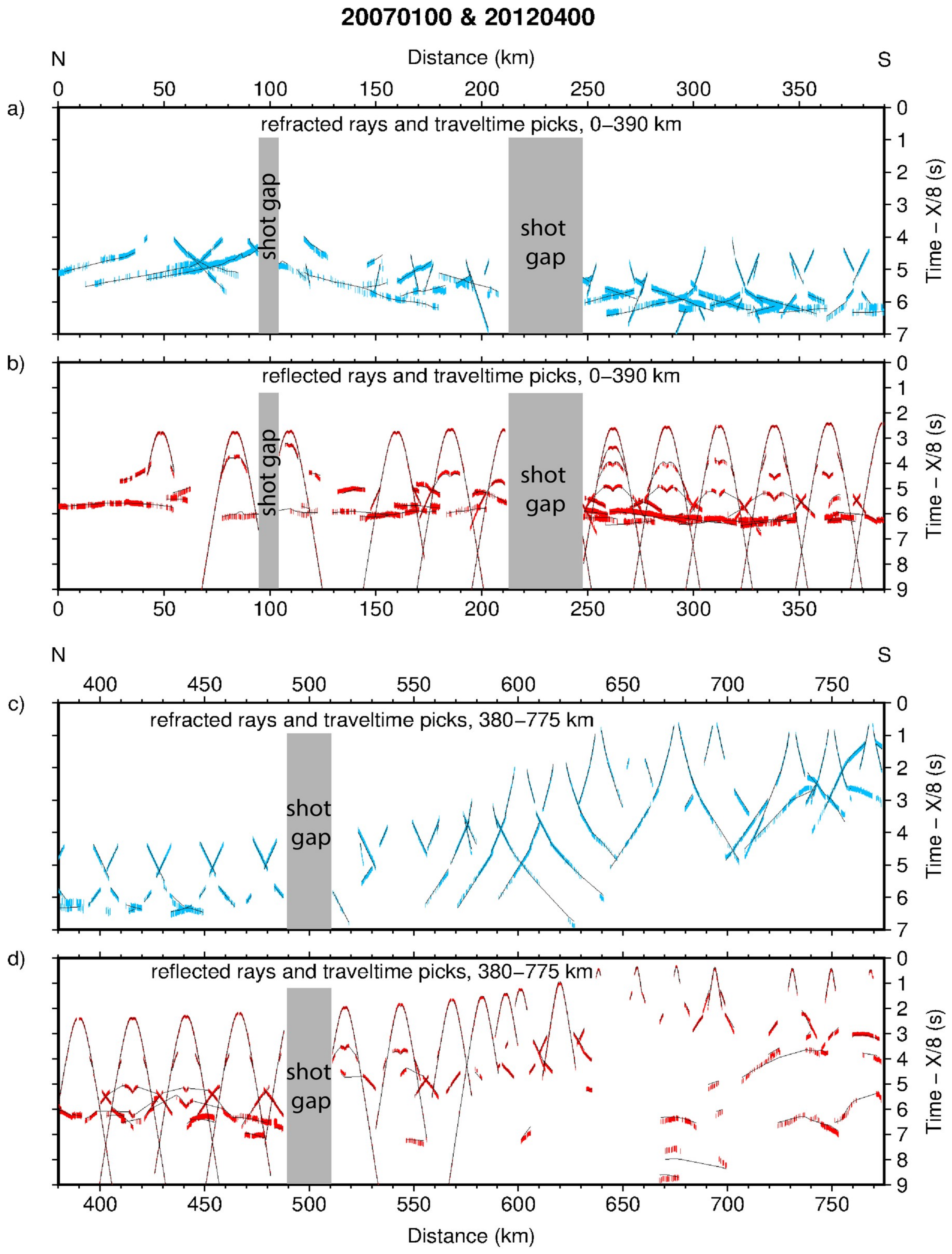

Supplementary Figure S10: Merged profiles 20070100 and 20120400: Quality of modelled picks. Because of the length of the profile the modelling results are split into two panels with a minor overlap around profile kilometer 380-390, respectively. For both, the reflected and refracted picks,

their length represents the assigned travel time error. Black lines represent modelled travel times. Shot gaps are highlighted in grey.

(a) the blue bars show the picks of the refracted arrivals with their error bars for the northern part of the transect (km 0-390; deep sea).

(b) the red bars show the picks of the reflected arrivals with their error bars for the northern part of the transect (km 0-390; deep sea).

(c) the blue bars show the picks of the refracted arrivals with their error bars for the southern part of the transect (km 380-775; transition to the Prydz Bay shelf area).

(d) the red bars show the picks of the reflected arrivals with their error bars for the southern part of the transect (km 380-775; transition to the Prydz Bay shelf area).

(5) Summary Table on deep seismic data acquisition and modelling

Name of profile	20170300	20120400	20070100	20070200
OBS	30	11	19	13
OBH	-	-	3	2
landstations	15	-	-	-
Number airguns	8	18 / 2	8 / 2	10
Total volume of airgun array	68 l	30-47 l / 60 l	68 l / 40 l	101 l
Towed depth of airgun array	10 m	5 m / 10 m	10 m / 10 m	13 m
Shot interval	60 sec	60 sec / 60 sec	60 sec / 60 sec	60 sec
Sampling frequency (OBS)	250 Hz	250 Hz	250 Hz	250 Hz

Name of P-wave velocity model	20170300	20120400 & 20070100	20070200
P-wave velocity model length	509 km	774 km	399 km
Number of data points used	~20000	~16600	~9500
RMS traveltime residual	76 ms	59 ms	62 ms
Normalized chi-squared	0.845	0.808	0.899
Assigned pick-uncertainties	60 – 105 ms	45 – 110 ms	45 – 80 ms

Supplementary Table S11: Summary table for the three P-wave velocity models . Upper panel: data acquisition parameter, lower panel: modeling parameters describing the quality of the velocity-depth models.
